# COVID‐19 and biodiversity: The paradox of cleaner rivers and elevated extinction risk to iconic fish species

**DOI:** 10.1002/aqc.3416

**Published:** 2020-06-19

**Authors:** Adrian C. Pinder, Rajeev Raghavan, J. Robert Britton, Steven J. Cooke

**Affiliations:** ^1^ Faculty of Science and Technology Bournemouth University Dorset UK; ^2^ Mahseer Trust c/o Freshwater Biological Association, Wareham Dorset UK; ^3^ Department of Fisheries Resource Management Kerala University of Fisheries and Ocean Studies (KUFOS) Kochi India; ^4^ South Asia Office IUCN SSC/WI Freshwater Fish Specialist Group (FFSG) Coimbatore India; ^5^ Fish Ecology and Conservation Physiology Laboratory, Department of Biology Carleton University Ottawa ON Canada

**Keywords:** COVID‐19, food security, India, mahseer, poaching, *Tor remadevii*

Notwithstanding the human suffering caused by COVID‐19, the response (e.g. shelter‐in‐place orders) has yielded some tangible environmental benefits such as substantial improvements in air and water quality (Corlett et al., 2020). In India, this has manifested as heavily polluted rivers now running clear for the first time in decades with, for example, reports suggesting that the quality of the River Ganges has improved sufficiently to support safe bathing.

Hidden beneath these brighter stories however, COVID‐19 is also intensifying pressure on India's aquatic wildlife. In an already poverty‐stricken country, an additional ~12 million are predicted to face extreme poverty as a result of COVID‐19 (World Bank, 2020). Lacking social security, 90% of India's workforce are entirely dependent on daily wages, and are heavily reliant on food supply chains (Reardon et al., 2019) that have been severely disrupted across rural India. With fish (farmed as well as marine‐sourced) and meat forming a primary source of protein for many, its sudden unavailability has resulted in local communities exploiting wild populations, especially freshwater fish.

**FIGURE 1:**
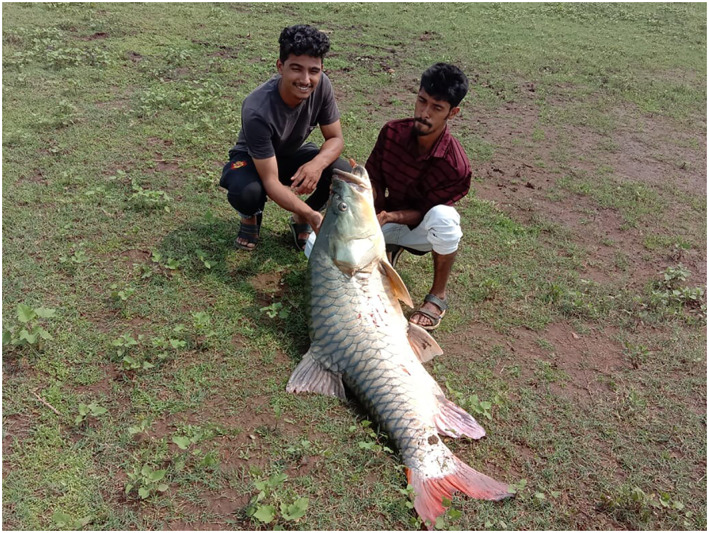
Youths with a Critically Endangered hump‐backed mahseer, *Tor remadevii*, caught from the Harangi Reservoir in Kodagu, Karnataka, India [Photo Credit: Star of Mysore]

As most newly recruited fishers lack knowledge on responsible and regulated capture techniques, illegal, indiscriminate and destructive methods are being used that have impacts on all aquatic fauna (e.g. dynamite, poisons). This also includes harvesting species of high extinction risk, exemplified by the endemic hump‐backed mahseer (*Tor remadevii*, Figure 1), an iconic and critically endangered member of the freshwater megafauna (Pinder, Raghavan, & Britton, in press) symbolic of India's extraordinarily diverse aquatic life. There is increasing evidence that their last remaining giant specimens are being removed from South India's River Cauvery by illegal fishers using a variety of capture methods (Deccan Herald, 2020), pushing them a step closer to extinction. This demonstrates that to understand fully the longer‐term environmental impacts of COVID‐19, there is always a need to look beneath the surface.
